# Insulin resistance-related surrogate indices and recurrence of mild hypertriglyceridemic acute pancreatitis: a retrospective cohort study

**DOI:** 10.3389/fnut.2026.1875732

**Published:** 2026-06-30

**Authors:** Zilong Yue, Yi Tang, Yarong Wang, Zhe Wang, Ruguang Xi, Long Qian

**Affiliations:** 1 General Surgery Department, Guoyang County People's Hospital, Bozhou, Anhui, People's Republic of China; 2 General Surgery Department, Wuhu Hospital of Traditional Chinese Medicine, Wuhu, Anhui, People's Republic of China; 3 Department of Neurology, The Fifth Clinical School of Medicine, Anhui Medical University, Hefei, Anhui, People's Republic of China; 4 Department of Geriatric, General Hospital of Ningxia Medical University, Yinchuan, Ningxia Hui Autonomous Region, People's Republic of China; 5 Department of Clinical Medicine, Hubei Enshi College, Enshi, Hubei, People's Republic of China

**Keywords:** hypertriglyceridemic acute pancreatitis, insulin resistance, lipid metabolism disorders, METS-IR, recurrence, TG/HDL-C, TyG index, TyG-BMI

## Abstract

**Background:**

Surrogate indices related to insulin resistance (IR) may reflect the metabolic abnormalities associated with hypertriglyceridemic acute pancreatitis (HAP). However, their association with disease recurrence remains unclear.

**Methods:**

A total of 412 eligible patients with HAP were included. Insulin resistance (IR) was assessed using the triglyceride-glucose index (TyG index), triglyceride-glucose-body mass index (TyG-BMI), metabolic score for insulin resistance (METS-IR), and triglyceride/high-density lipoprotein cholesterol ratio (TG/HDL-C). Associations with HAP recurrence were examined using multivariable logistic regression and restricted cubic spline (RCS) analyses. Predictive and reclassification performances were evaluated using receiver operating characteristic (ROC) curves, net reclassification improvement (NRI), integrated discrimination improvement (IDI), and false discovery rate (FDR) correction.

**Results:**

The cumulative recurrence rate of HAP was 41.26%. Following comprehensive covariate adjustment, individuals in the highest quartiles of TyG-BMI (OR = 2.70, 95% CI: 1.42–5.20), METS-IR (OR = 2.62, 95% CI: 1.37–5.06), and TG/HDL-C (OR = 2.21, 95% CI: 1.17–4.22) exhibited a significantly elevated risk of recurrence. These principal associations remained robust after FDR correction. Additionally, RCS modeling confirmed significant dose-response trajectories for the TyG index, TyG-BMI, and METS-IR. Among the evaluated indices, TyG-BMI yielded the numerically highest AUC (0.613). Moreover, discordant outcomes in NRI and IDI analyses relative to alternative indices suggested that no single marker holds absolute predictive superiority.

**Conclusion:**

Baseline insulin resistance-related surrogate indices are independently associated with the recurrence of mild HAP. These routine and accessible parameters may serve as practical prognostic indicators for long-term recurrence risk.

## Introduction

Acute pancreatitis (AP) has a multifactorial and complex pathogenesis, with a high incidence and hospitalization rate, placing a substantial health and economic burden on healthcare systems globally (1–3). One frequent subtype of AP is hypertriglyceridemic acute pancreatitis (HAP), which is characterized by a notable increase in serum triglyceride (TG) levels (4). The hydrolysis of excess TGs into free fatty acids (FFAs) induces pancreatic and peripheral microthrombosis, thereby disrupting local microcirculation. Although most patients with HAP present with mild and self-limiting symptoms, HAP is prone to recurrence due to its distinct pathophysiology (5). Recurrent episodes of AP can result in permanent pancreatic damage, which may eventually progress to chronic pancreatitis, severely affecting both the quality of life and imposing a heavy economic burden on patients. Furthermore, repeated episodes of AP significantly increase the risk of developing pancreatic cancer (6).

Lipid metabolism disorders are widely recognized as key contributors to the development of HAP, as these disorders lead to an increase in pro-inflammatory mediators, exacerbating tissue damage and necrosis (7, 8). One critical factor that significantly contributes to the pathogenesis of lipid metabolism disorders is insulin resistance (IR). IR is an abnormality in insulin receptors that renders insulin ineffective in lowering blood glucose, which leads to an excess release and accumulation of insulin in the body (9). Disordered lipid metabolism is both a consequence and a driver of IR, with these conditions having a reciprocal relationship. Previous research demonstrated that elevated triglycerides can induce IR, which in turn worsens lipid metabolism disorders (10).

In clinical studies where direct insulin measurements are unavailable, several metabolic indices derived from routine clinical parameters have been used as surrogate markers of insulin resistance related metabolic dysfunction. These include the triglyceride-glucose (TyG) index, triglyceride-glucose body mass index (TyG-BMI), metabolic score for insulin resistance (METS-IR), and triglyceride to high-density lipoprotein cholesterol (TG/HDL-C) ratio. Among these indices, the TyG index has been widely investigated and shown to be associated with various cardiometabolic conditions, including acute and chronic coronary syndromes, hypertension, non-alcoholic fatty liver disease, and diabetes (11–18). However, whether these surrogate indices are associated with the recurrence of HAP remains unclear.

Given the biological links among dyslipidemia, insulin resistance-related metabolic dysfunction, and HAP, we evaluated the associations of the TyG index, TyG-BMI, METS-IR, and TG/HDL-C with the risk of HAP recurrence.

## Methods

### Study design

Patients diagnosed with HAP at Wuhu Hospital of Traditional Chinese Medicine and the Guoyang Branch of Anhui Provincial Hospital between January 1, 2016, and April 30, 2021, were enrolled in this study. All included patients were evaluated for recurrence over a standardized period of 24 months following discharge from their index episode. At the 2-year follow-up, recurrence status was assessed by determining whether any recurrent HAP episode had occurred during the 24-month period after discharge. Consequently, the primary outcome was defined as HAP recurrence within 2 years after discharge.

The diagnostic criteria for mild HAP comprised: (1) mild AP according to the 2012 Atlanta classification, which requires at least two typical features (characteristic abdominal pain, pancreatic enzymes ≥ 3 times the upper limit of normal, or positive imaging findings) without organ failure or complications (19); (2) exclusion of alternative etiologies; and (3) a serum triglyceride level ≥ 11.3 mmol/L. For episodes occurring within our centers, relevant diagnoses were confirmed by reviewing computed tomography scans retrieved from the electronic medical record (EMR) system. In contrast, external recurrences were captured through telephone interviews based on patient self-reports of CT-verified conditions.

Baseline demographic characteristics and laboratory indices were collected at the time of the first HAP episode. The following surrogate indices were calculated: TyG index = ln [TG (mg/dL) × fasting blood glucose (FBG, mg/dL)/2]; TyG-BMI = TyG index × BMI; METS-IR = ln [(2 × FBG (mg/dL)) + TG (mg/dL)] × BMI/ln [HDL-C (mg/dL)]; and TG/HDL-C = TG (mg/dL)/HDL-C (mg/dL). These indices were subsequently examined in relation to HAP recurrence within 2 years after discharge to assess their potential value for baseline risk assessment after the index episode.

This study obtained approval from the Ethics Committee of the Wuhu Hospital of Traditional Chinese Medicine and Guoyang Branch of Anhui Provincial Hospital. Since this was a retrospective study, it obtained a written waiver of consent approved by the Ethics Committee.

The inclusion criteria were: (1) admission to the hospital within 48 h of symptom onset; and (2) a confirmed diagnosis of mild HAP. The exclusion criteria comprised: (1) incomplete or missing baseline clinical data; (2) loss to follow-up; and (3) death from causes unrelated to HAP during the follow-up period.

### Outcome

Patients were stratified into two groups based on the occurrence of HAP recurrence during the follow-up period. The primary objective of this study was to investigate the associations between baseline IR-related surrogate indices and the risk of 2-year HAP recurrence.

### Statistical analysis

Continuous variables were summarized as means (SDs) and compared using the *t*-test, while categorical variables were summarized as frequencies and percentages and compared using the chi-square test. Univariable logistic regression was first employed to examine preliminary associations with HAP recurrence. Three covariate-adjusted logistic regression models were subsequently constructed to evaluate the independent associations between IR-related surrogate indices and HAP recurrence. Restricted cubic spline (RCS) analysis was implemented to characterize potential non-linear associations. Model discrimination and reclassification capabilities were rigorously assessed using receiver operating characteristic curves, the areas under the curve (AUCs), continuous net reclassification improvement (NRI), and integrated discrimination improvement (IDI). Furthermore, false discovery rate (FDR) corrections were applied to account for multiple comparisons. All *P* values were two-sided, and a *P* < 0.05 was considered statistically significant. All statistical analyses were conducted using R version 4.2.1.

## Results

### Baseline characteristics

**Figure 1:**
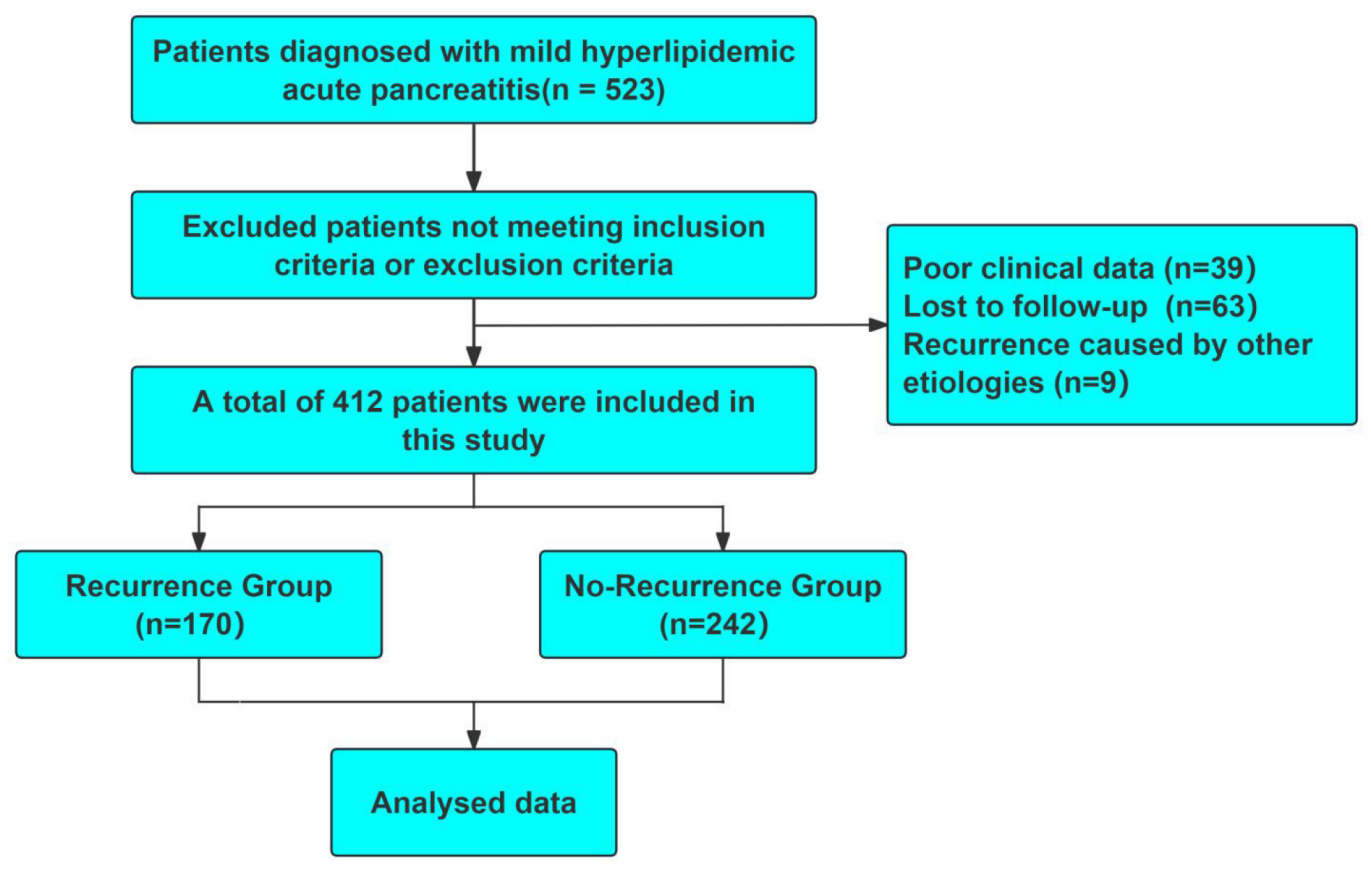
Flow chart.

A total of 412 patients with HAP were included in the analysis, of whom 326 were male and 86 were female. The recurrence rate of HAP was 41.26%. The flowchart is shown in Figure 1. The Recurrence group included 170 patients (141 males, 29 females), with a mean age of 31.55 ± 9.01 years. Meanwhile, the No-Recurrence group comprised 242 patients (185 males, 57 females), with a mean age of 30.25 ± 7.68 years. The two groups differed significantly in BMI, white blood cell count (WBC), amylase (AMS), TG, TyG index, and METS-IR (all *P* < 0.05), with details presented in Table 1.

**Table 1:** Baseline characteristics.

Variables	Total (*n* = 412)	No-recurrence (*n* = 242)	Recurrence (*n* = 170)	*P*
Age, years	30.79 ± 8.27	30.25 ± 7.68	31.55 ± 9.01	0.128
Sex				
Male	326 (79.13)	185 (76.45)	141 (82.94)	0.110
Female	86 (20.87)	57 (23.55)	29 (17.06)	
T2DM				
No	378 (91.75)	226 (93.39)	152 (89.41)	0.149
Yes	34 (8.25)	16 (6.61)	18 (10.59)	
Hypertension				
No	383 (92.96)	227 (93.80)	156 (91.76)	0.426
Yes	29 (7.04)	15 (6.20)	14 (8.24)	
BMI, kg/m^2^	29.34 ± 3.13	28.96 ± 3.16	29.89 ± 3.02	0.003
WBC, ^*^10^9^/L	12.37 ± 3.19	12.05 ± 3.01	12.81 ± 3.39	0.017
NEUT, ^*^10^9^/L	9.94 ± 3.06	9.94 ± 3.07	9.94 ± 3.06	0.993
RBC, ^*^10^12^/L	4.67 ± 0.40	4.65 ± 0.39	4.70 ± 0.41	0.231
HB, g/L	142.18 ± 17.60	141.14 ± 17.94	143.65 ± 17.05	0.154
PLT, ^*^10^9^/L	212.34 ± 41.86	210.49 ± 40.53	214.97 ± 43.68	0.285
ALB, g/L	39.84 ± 4.93	40.02 ± 4.95	39.59 ± 4.91	0.374
AMS, U/L	434.04 ± 200.28	405.86 ± 190.39	474.16 ± 207.61	< 0.001
TBIL, umol/L	14.99 ± 5.06	14.90 ± 5.06	15.10 ± 5.08	0.692
TC, mmol/L	14.84 ± 5.05	14.70 ± 4.83	15.03 ± 5.36	0.519
TG, mmol/L	19.29 ± 4.59	18.49 ± 4.45	20.44 ± 4.55	< 0.001
HDL-C, mmol/L	0.84 ± 0.31	0.84 ± 0.31	0.84 ± 0.31	0.846
LDL-C, mmol/L	1.65 ± 0.88	1.66 ± 0.91	1.64 ± 0.84	0.815
FBG, mmol/L	7.10 ± 2.59	6.93 ± 2.36	7.34 ± 2.88	0.111
Potassium, mmol/L	4.54 ± 0.48	4.51 ± 0.46	4.58 ± 0.51	0.149
Sodium, mmol/L	129.02 ± 5.34	129.10 ± 5.30	128.91 ± 5.40	0.720
Calcium, mmol/L	2.28 ± 0.64	2.30 ± 0.64	2.26 ± 0.64	0.557
ALT, U/L	28.07 ± 8.80	28.01 ± 8.97	28.15 ± 8.57	0.876
AST, U/L	50.97 ± 19.37	50.79 ± 19.17	51.21 ± 19.70	0.829
PT, s	12.03 ± 1.51	12.10 ± 1.58	11.94 ± 1.40	0.287
TyG index	11.52 ± 0.44	11.45 ± 0.44	11.61 ± 0.42	< 0.001
TG/HDL-C	62.05 ± 33.78	60.33 ± 35.41	64.50 ± 31.25	0.218
METS-IR	66.29 ± 12.31	65.25 ± 12.43	67.77 ± 12.01	0.040
TyG-BMI	338.14 ± 40.25	331.85 ± 40.46	347.09 ± 38.31	< 0.001

**Data:** *N* (%) or Mean ± Standard Deviation.

BMI, body mass index; WBC, white blood cell; NEUT, neutrophil; RBC, red blood cell; HB, hemoglobin; PLT, blood platelet; ALB, albumin; AMS, blood amylase; TBIL, total bilirubin; TC, total cholesterol; TG, triglyceride; HDL-C, high-density lipoprotein cholesterol; LDL-C, low-density lipoprotein cholesterol; FBG, fasting blood glucose; TyG index, triglyceride-glucose index; ALT, alanine aminotransferase; AST, aspartate aminotransferase; PT, prothrombin time.

### Univariate logistic regression analysis of recurrence of hypertriglyceridemic acute pancreatitis

Univariable logistic regression analysis revealed that the TyG index (OR = 2.27, 95% CI: 1.43–3.61, *P* < 0.001), TyG-BMI (OR = 1.01, 95% CI: 1.00–1.01, *P* < 0.001), and METS-IR (OR = 1.02, 95% CI: 1.01–1.03, *P* = 0.042) were significantly associated with an increased risk of HAP recurrence. In contrast, the TG/HDL-C ratio did not exhibit a statistically significant association (OR = 1.00, 95% CI: 1.00–1.01, *P* = 0.219). Other baseline variables significantly associated with recurrence included BMI, WBC, AMS, and TG (Table 2).

**Table 2:** Univariable logistic regression analysis of HAP recurrence.

Variables	β	S.E	*Z*	*P*	OR (95%CI)
Sex					
Female					Reference
Male	0.40	0.25	1.59	0.112	1.50 (0.91–2.46)
T2DM					
No					Reference
Yes	0.51	0.36	1.43	0.152	1.67 (0.83–3.38)
Hypertension					
No					Reference
Yes	0.31	0.39	0.79	0.428	1.36 (0.64–2.89)
Age, years	0.02	0.01	1.56	0.118	1.02 (1.00–1.04)
BMI, kg/m^2^	0.10	0.03	2.97	0.003	1.10 (1.03–1.17)
WBC, ^*^10^9^/L	0.07	0.03	2.37	0.018	1.08 (1.01–1.15)
NEUT, ^*^10^9^/L	0.00	0.03	0.01	0.993	1.00 (0.94–1.07)
RBC, ^*^10^12^/L	0.30	0.25	1.20	0.230	1.35 (0.83–2.21)
HB, g/L	0.01	0.01	1.43	0.154	1.01 (1.00–1.02)
PLT, ^*^10^9^/L	0.00	0.00	1.07	0.285	1.00 (1.00–1.01)
ALB, g/L	−0.02	0.02	−0.89	0.373	0.98 (0.94–1.02)
AMS, U/L	0.01	0.00	3.37	< 0.001	1.01 (1.01–1.01)
TBIL, umol/L	0.01	0.02	0.40	0.691	1.01 (0.97–1.05)
TC, mmol/L	0.01	0.02	0.65	0.518	1.01 (0.97–1.05)
TG, mmol/L	0.10	0.02	4.17	< 0.001	1.10 (1.05–1.15)
HDL-C, mmol/L	0.06	0.32	0.19	0.846	1.06 (0.57–2.00)
LDL-C, mmol/L	−0.03	0.11	−0.23	0.815	0.97 (0.78–1.22)
FBG, mmol/L	0.06	0.04	1.59	0.112	1.06 (0.99–1.15)
Potassium, mmol/L	0.30	0.21	1.44	0.149	1.35 (0.90–2.04)
Sodium, mmol/L	−0.01	0.02	−0.36	0.719	0.99 (0.96–1.03)
Calcium, mmol/L	−0.10	0.20	−0.59	0.556	0.90 (0.66–1.22)
ALT, U/L	0.00	0.01	0.16	0.875	1.00 (0.98–1.02)
AST, U/L	0.00	0.01	0.22	0.828	1.00 (0.99–1.01)
PT, s	−0.07	0.07	−1.07	0.286	0.93 (0.82–1.06)
TyG	0.82	0.24	3.48	< 0.001	2.27 (1.43–3.61)
TG/HDL-C	0.00	0.00	1.23	0.219	1.00 (1.00–1.01)
METS-IR	0.02	0.01	2.04	0.042	1.02 (1.01–1.03)
TyG-BMI	0.01	0.00	3.74	< 0.001	1.01 (1.0–1.01)

BMI, body mass index; WBC, white blood cell; NEUT, neutrophil; RBC, red blood cell; HB, hemoglobin; PLT, blood platelet; ALB, albumin; AMS, blood amylase; TBIL, total bilirubin; TC, total cholesterol; TG, triglyceride; HDL-C, high-density lipoprotein cholesterol; LDL-C, low-density lipoprotein cholesterol; FBG, fasting blood glucose; TyG index, triglyceride-glucose index; ALT, alanine aminotransferase; AST, aspartate aminotransferase; PT, prothrombin time.

### TyG index and risk of HAP recurrence

In Model 1, patients in Q4 of the TyG index exhibited significantly higher odds of HAP recurrence than those in Q1 (OR = 2.45, 95% CI: 1.39–4.35, *P* = 0.002). Following full covariate adjustment in Model 3, this association remained statistically significant (OR = 2.08, 95% CI: 1.07–4.10, *P* = 0.033), as shown in Table 3.

**Table 3:** Association between IR-related indicators and the recurrence of HAP.

Exposure	Model 1		Model 2		Model 3	
	OR (95%CI)	*P*	OR (95%CI)	*P*	OR (95%CI)	*P*
TyG index						
Q 1	1.00 (Reference)		1.00 (Reference)		1.00 (Reference)	
Q 2	1.53 (0.86–2.72)	0.147	1.46 (0.82–2.63)	0.199	1.38 (0.75–2.56)	0.301
Q 3	1.65 (0.94–2.95)	0.084	1.40 (0.78–2.52)	0.265	1.24 (0.66–2.33)	0.498
Q 4	2.45 (1.39–4.35)	0.002	2.31 (1.28–4.21)	0.006	2.08 (1.07–4.10)	0.033
TyG-BMI						
Q 1	1.00 (Reference)		1.00 (Reference)		1.00 (Reference)	
Q 2	1.25 (0.70–2.27)	0.454	1.29 (0.71–2.36)	0.401	1.21 (0.65–2.26)	0.557
Q 3	2.58 (1.46–4.62)	0.001	2.71 (1.50–4.98)	0.001	2.62 (1.37–5.06)	0.004
Q 4	2.48 (1.41–4.44)	0.002	2.59 (1.45–4.70)	0.001	2.70 (1.42–5.20)	0.003
TG/HDL-C						
Q 1	1.00 (Reference)		1.00 (Reference)		1.00 (Reference)	
Q 2	1.57 (0.90–2.79)	0.116	1.65 (0.93–2.94)	0.089	1.86 (1.01–3.44)	0.047
Q 3	1.34 (0.76–2.38)	0.311	1.51 (0.84–2.74)	0.170	1.66 (0.89–3.12)	0.112
Q 4	1.91 (1.09–3.38)	0.024	2.12 (1.19–3.82)	0.012	2.21 (1.17–4.22)	0.015
METS-IR						
Q 1	1.00 (Reference)		1.00 (Reference)		1.00 (Reference)	
Q 2	1.59 (0.90–2.83)	0.112	1.78 (0.99–3.23)	0.054	1.50 (0.81–2.81)	0.195
Q 3	1.59 (0.90–2.83)	0.112	1.77 (0.98–3.21)	0.059	1.53 (0.82–2.89)	0.182
Q 4	2.26 (1.29–4.02)	0.005	2.67 (1.48–4.89)	0.001	2.62 (1.37–5.06)	0.004

Model 1: Crude.

Model 2: Adjust: Sex, T2DM, Hypertension, Age.

Model 3: Adjust: Sex, T2DM, Hypertension, Age, WBC, NEUT, RBC, HB, PLT, ALB, AMS, TBIL, Potassium, Sodium, Calcium, ALT, AST, PT.

### TyG-BMI and risk of HAP recurrence

In Model 1, compared with Q1, TyG-BMI Q3 was significantly associated with higher odds of HAP recurrence (OR = 2.58, 95% CI: 1.46–4.62, *P* = 0.001), and a similar elevation in risk was observed for Q4 (OR = 2.48, 95% CI: 1.41–4.44, *P* = 0.002). After full covariate adjustment in Model 3, independent associations persisted for both Q3 (OR = 2.62, 95% CI: 1.37–5.06, *P* = 0.004) and Q4 (OR = 2.70, 95% CI: 1.42–5.20, *P* = 0.003), as presented in Table 3.

### TG/HDL-C and risk of HAP recurrence

Regarding the TG/HDL-C ratio, Model 1 demonstrated that patients in Q4 had significantly higher odds of HAP recurrence compared with those in Q1 (OR = 1.91, 95% CI: 1.09–3.38, *P* = 0.024). This significant association persisted in Model 3 after full covariate adjustment (OR = 2.21, 95% CI: 1.17–4.22, *P* = 0.015).

### METS-IR and risk of HAP recurrence

Model 1 showed that patients in Q4 had significantly higher odds of HAP recurrence than those in Q1 (OR = 2.26, 95% CI: 1.29–4.02, *P* = 0.005). Following full covariate adjustment in Model 3, this independent association remained statistically robust (OR = 2.62, 95% CI: 1.37–5.06, *P* = 0.004).

### E-value analysis

E-value analysis was performed for Q4 vs. Q1 comparisons (Table 4). The E-values for the point estimates (and the corresponding lower 95% CIs) were 2.24 (1.22) for the TyG index, 2.67 (1.67) for TyG-BMI, 2.34 (1.38) for TG/HDL-C, and 2.62 (1.62) for METS-IR.

**Table 4:** E-value sensitivity analysis for unmeasured confounding (Q4 vs. Q1).

Indicators	E-value (point estimate)	E-value (lower 95% CI)
TyG index	2.24	1.22
TyG-BMI	2.67	1.67
TG/HDL-C	2.34	1.38
METS-IR	2.62	1.62

### FDR correction for multiple comparisons

After FDR correction for Model 3 quartile comparisons, the associations of TyG-BMI Q3 and Q4 with HAP recurrence remained statistically significant, with FDR-adjusted *P* values of 0.015 and 0.015, respectively. The associations also remained significant for TG/HDL-C Q4 and METS-IR Q4, with FDR-adjusted *P* values of 0.046 and 0.015, respectively. In contrast, the association for TyG index Q4 did not remain significant after FDR correction (*P* = 0.079), detailed in Table 5.

**Table 5:** FDR correction for Model 3 quartile comparisons.

Exposure	OR (95%CI)	P	FDR
TyG index			
Q1	1.00 (Reference)		
Q2	1.38 (0.75–2.56)	0.301	0.361
Q3	1.24 (0.66–2.33)	0.498	0.543
Q4	2.08 (1.07–4.10)	0.033	0.079
TyG-BMI			
Q1	1.00 (Reference)		
Q2	1.21 (0.65–2.26)	0.557	0.557
Q3	2.62 (1.37–5.06)	0.004	0.015
Q4	2.70 (1.42–5.20)	0.003	0.015
TG/HDL-C			
Q1	1.00 (Reference)		
Q2	1.86 (1.01–3.44)	0.047	0.094
Q3	1.66 (0.89–3.12)	0.112	0.192
Q4	2.21 (1.17–4.22)	0.015	0.046
METS-IR			
Q1	1.00 (Reference)		
Q2	1.50 (0.81–2.81)	0.195	0.260
Q3	1.53 (0.82–2.89)	0.182	0.260
Q4	2.62 (1.37–5.06)	0.004	0.015

This model adjusts for Sex, T2DM, Hypertension, Age, WBC, NEUT, RBC, HB, PLT, ALB, AMS, TBIL, Potassium, Sodium, Calcium, ALT, AST, PT.

### Dose-response relationships between IR-related indices and HAP Recurrence

**Figure 2:**
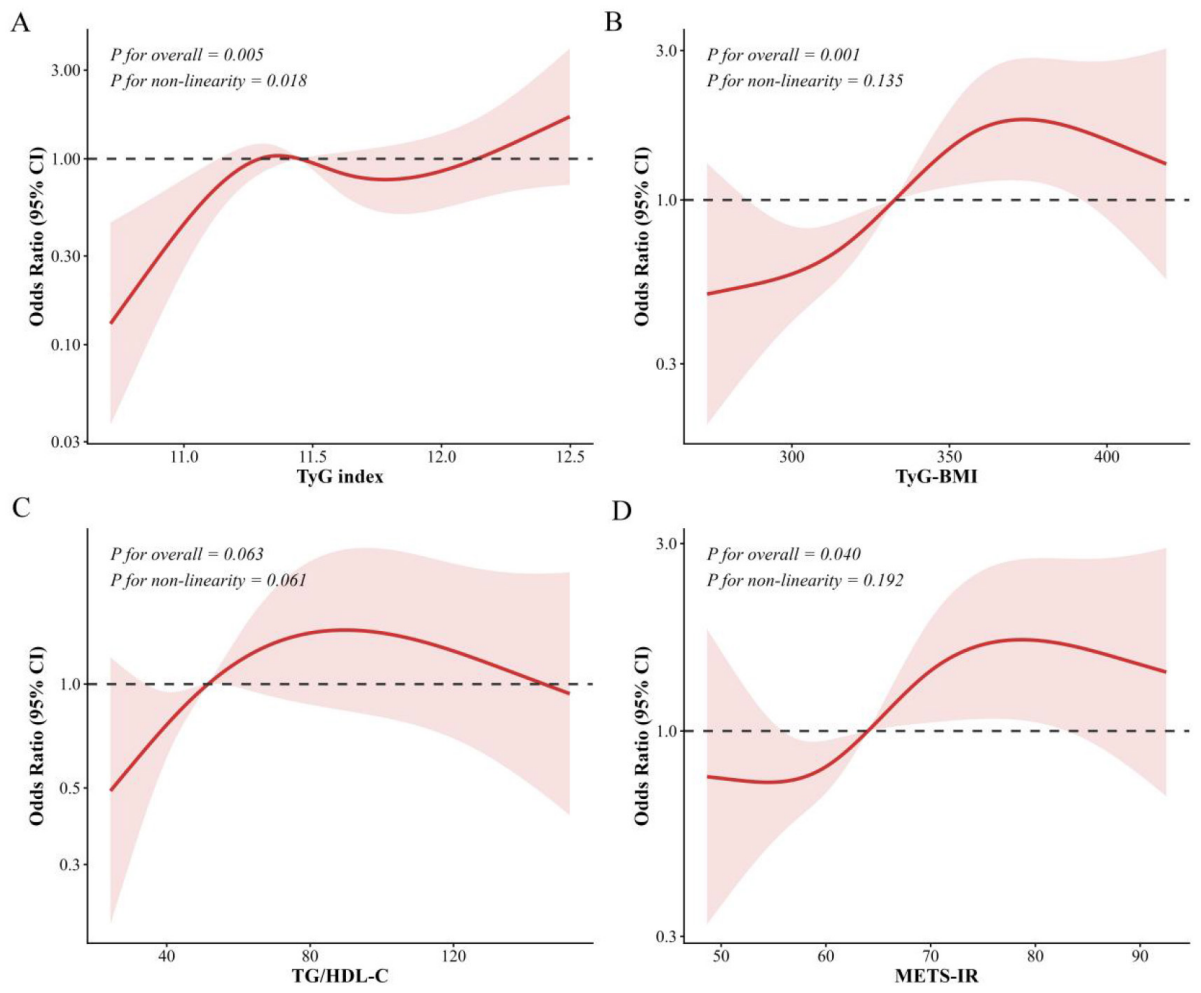
Restricted cubic spline models showing the associations between IR–related indices and recurrence of HAP. The models were adjusted for Sex, T2DM, Hypertension, Age, WBC, NEUT, RBC, HB, PLT, ALB, AMS, TBIL, Potassium, Sodium, Calcium, ALT, AST, PT. **(A–D)** Dose–response relationships between four insulin resistance–related indices and the risk of recurrence of hypertriglyceridemic acute pancreatitis: **(A)** TyG index, **(B)** TyG-BMI, **(C)** TG/HDL-C, and **(D)** METS-IR. The solid red lines represent the adjusted odds ratios (ORs), and the shaded areas indicate 95% confidence intervals (CIs). The horizontal dashed line represents the reference value (OR = 1.0).P for overall indicates the statistical significance of the overall association, and P for non-linearity tests whether the relationship deviates from linearity.

The TyG index was significantly associated with HAP recurrence, with a non-linear relationship (overall *P* = 0.005; *P* for non-linearity = 0.018). TyG-BMI was also significantly associated with HAP recurrence, with an approximately linear relationship (overall *P* = 0.001; *P* for non-linearity = 0.135). METS-IR showed a similar approximately linear association with HAP recurrence (overall *P* = 0.040; *P* for non-linearity = 0.192). In contrast, TG/HDL-C was not significantly associated with HAP recurrence in the RCS analysis (overall *P* = 0.063; *P* for non-linearity = 0.061). These findings are shown in Figure 2.

### Comparison of the predictive power of various IR-related Indices for HAP recurrence

**Figure 3:**
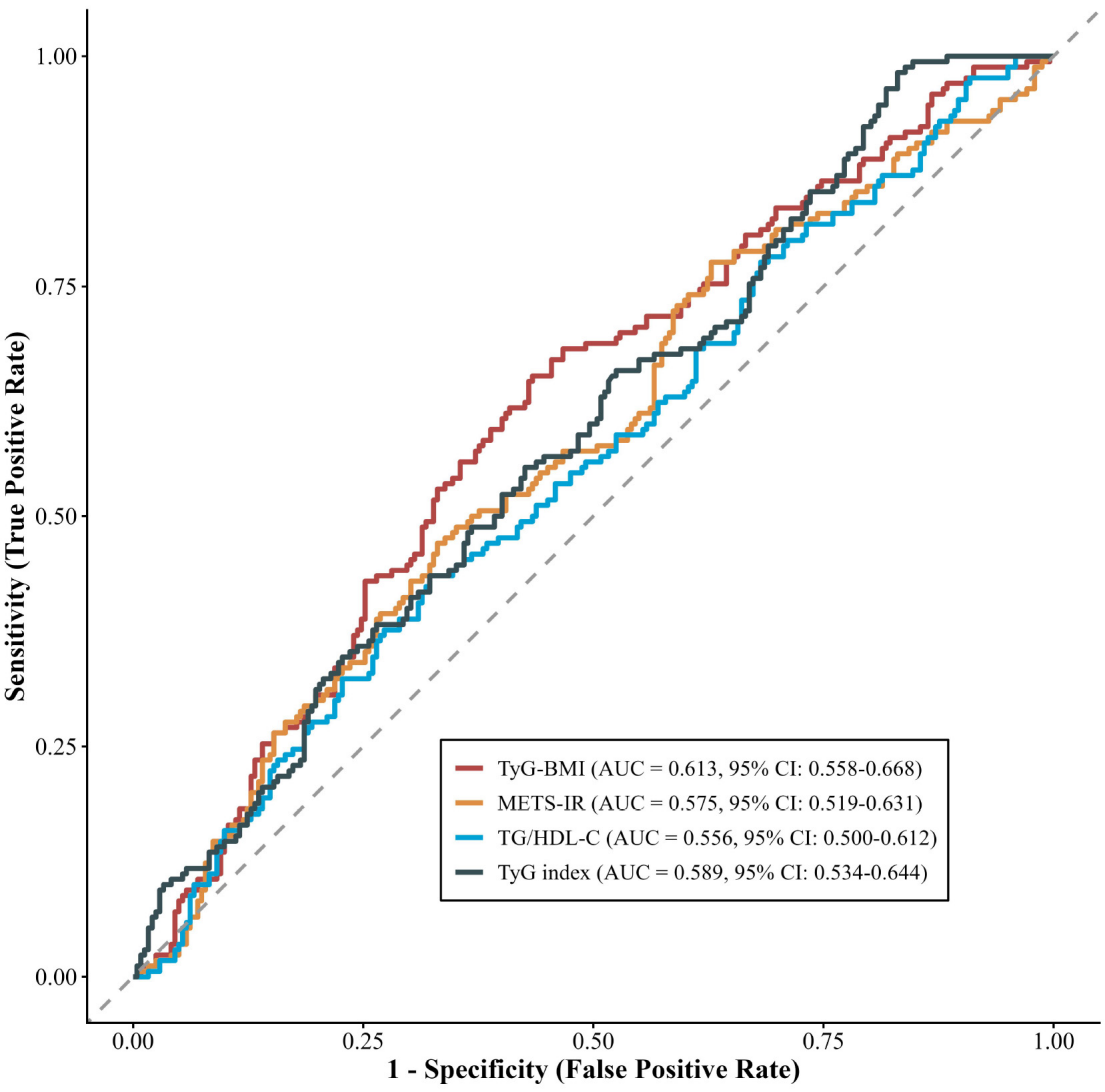
ROC curves of IR-related indices for HAP recurrence. ROC, receiver operating characteristic; AUC, area under the curve; CI, confidence interval; TyG, triglyceride-glucose; BMI, body mass index; TG/HDL-C, triglyceride to high-density lipoprotein cholesterol ratio; METS-IR, metabolic score for insulin resistance.

ROC curve analysis was performed to evaluate the discriminatory performance of different IR-related indices for HAP recurrence (Figure 3). The TyG-BMI index had an AUC of 0.613 (95% CI: 0.558–0.668), the TyG index 0.589 (95% CI: 0.534–0.644), METS-IR 0.575 (95% CI: 0.519–0.631), and TG/HDL-C 0.556 (95% CI: 0.500–0.612). The AUCs for the IR-related indices indicate their discriminatory performance for HAP recurrence. Among the evaluated indices, TyG-BMI had the numerically highest AUC, indicating the greatest discriminatory value among the four indices.

### Comparison of NRI and IDI among models based on IR-related indices

Tables 6, 7 summarize the NRI and IDI results for models based on different IR-related indices, using TyG-BMI as the reference model. Compared with TyG-BMI, the TyG index showed no significant difference in overall NRI (0.036, *P* = 0.716) or IDI (0.013, *P* = 0.115). METS-IR and TG/HDL-C had significantly lower overall NRI values than TyG-BMI (−0.343, *P* = 0.001; and −0.347, *P* < 0.001, respectively), suggesting poorer risk reclassification. However, both indices had significantly higher IDI values (0.016, *P* = 0.010; and 0.024, *P* = 0.004, respectively). These inconsistent NRI and IDI results suggest that no single index consistently outperformed the others across all predictive metrics.

**Table 6:** Comparison NRI among models based on IR-related indices.

Comparison models	NRIcomponent	NRI (95% CI)	*P*
TyG index vs. TyG-BMI	Overall NRI	0.036 (−0.159 to 0.232)	0.716
	Event NRI	0.094 (−0.056 to 0.244)	0.218
	Non-event NRI	−0.058 (−0.184 to 0.068)	0.367
METS-IR vs. TyG-BMI	Overall NRI	−0.343 (−0.536 to −0.150)	0.001
	Event NRI	−0.153 (−0.301 to −0.004)	0.044
	Non-event NRI	−0.190 (−0.314 to −0.066)	0.003
TG/HDL-C vs. TyG-BMI	Overall NRI	−0.347 (−0.540 to −0.153)	< 0.001
	Event NRI	−0.165 (−0.313 to −0.016)	0.029
	Non-event NRI	−0.182 (−0.306 to −0.058)	0.004

NRI, Net reclassification improvement. The TyG-BMI model was used as the reference model. Negative NRI values indicate poorer reclassification performance than the TyG-BMI model.

**Table 7:** Comparison IDI among models based on IR-related indices.

Comparison models	IDI	95% CI	*P*
TyG index vs. TyG-BMI	0.013	−0.003–0.029	0.115
METS-IR vs. TyG-BMI	0.016	0.004–0.028	0.010
TG/HDL-C vs TyG-BMI	0.024	0.008–0.040	0.004

### Subgroup analyses

**Figure 4:**
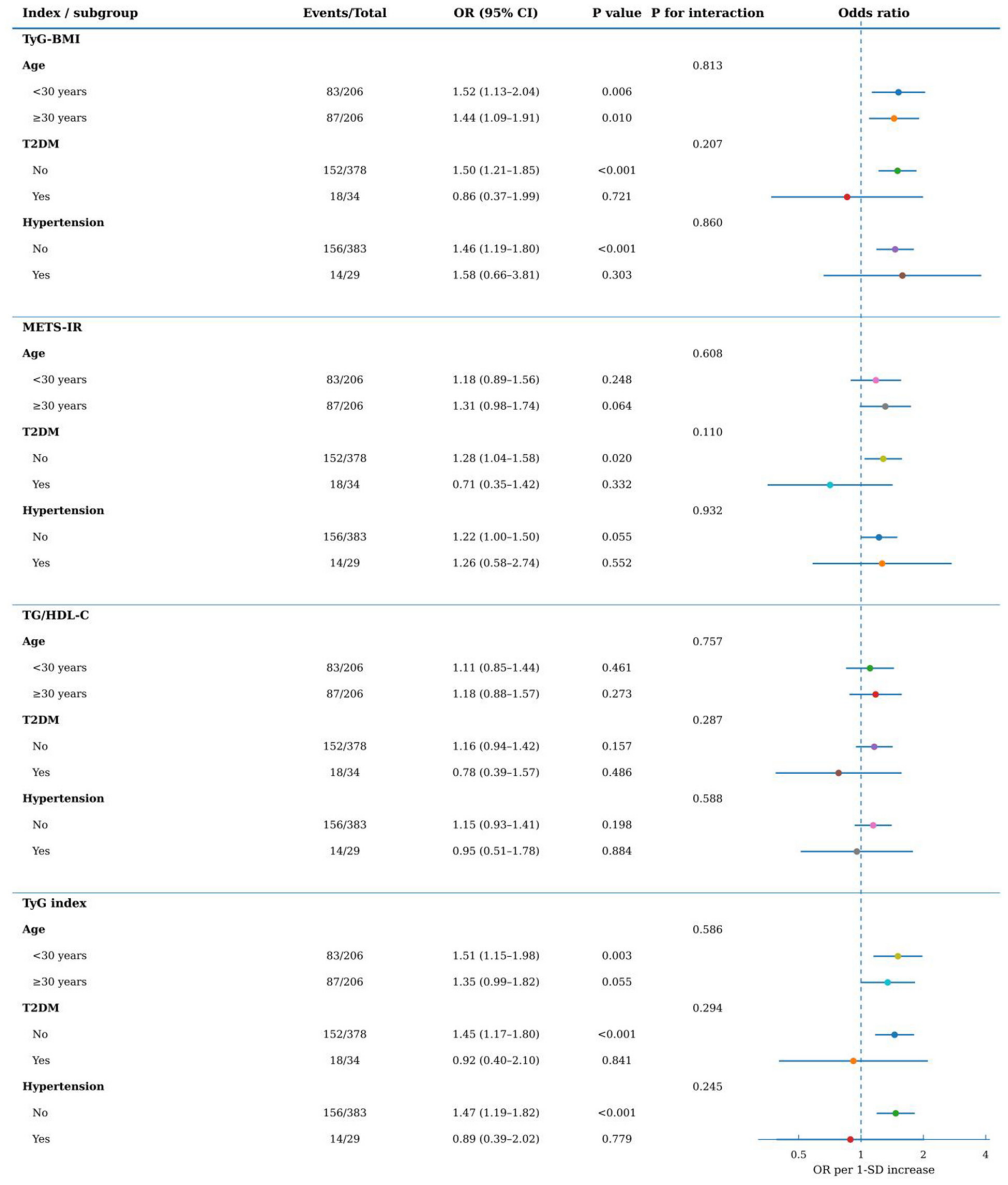
Subgroup analysis of IR-related indices and HAP recurrence. Subgroup analyses were stratified by baseline age, T2DM status, and hypertension status. Odds ratios (ORs) and 95% confidence intervals (CIs) are presented per 1-SD increase in each respective metabolic index. OR, odds ratio; CI, confidence interval; SD, standard deviation; T2DM, type 2 diabetes mellitus; TyG, triglyceride-glucose; BMI, body mass index; TG/HDL-C, triglyceride to high-density lipoprotein cholesterol ratio; METS-IR, metabolic score for insulin resistance.

Subgroup analyses stratified by age, T2DM status, and hypertension status are shown in Figure 4. The association between TyG-BMI and HAP recurrence was statistically significant in both age groups, as well as in patients without T2DM and those without hypertension, with generally consistent effect estimates across strata. A similar pattern was observed for the TyG index, whereas the associations for METS-IR and TG/HDL-C were less consistent. No significant interactions were observed between any IR-related index and age, T2DM status, or hypertension status.

## Discussion

In this retrospective cohort study, elevated levels of several insulin resistance–related indices were associated with an increased risk of HAP recurrence. Following multivariable adjustment and FDR correction, TyG-BMI and the TyG index remained significantly associated with recurrence. Among all evaluated indices, TyG-BMI demonstrated the most consistent association across subgroups and the highest AUC, with superior reclassification performance compared to other indices, including METS-IR and TG/HDL-C.

HAP is a subtype of AP and is closely associated with markedly elevated serum TG levels. Previous studies have reported recurrence rates of approximately 30.3%−32.9% in patients with HAP (20–22). In the present study population, the recurrence rate of HAP was 41.26%, suggesting a substantial risk of disease relapse. These findings indicate that HAP recurrence remains a significant clinical concern. Identifying simple and accessible biomarkers may help identify patients at an increased risk of recurrence and provide a basis for individualized follow-up and clinical management.

IR plays a critical role in lipid metabolism disorders, which are centrally involved in the pathogenesis of HAP. In clinical practice, the TyG index, TyG-BMI, TG/HDL-C, and METS-IR serve as readily accessible surrogate biomarkers of IR-related metabolic dysfunction (17, 23, 24). In the present study, elevated IR-related indices were associated with an increased risk of HAP recurrence, suggesting that IR-related metabolic abnormalities may drive disease relapse. Broadly speaking, this aligns with the fact that IR is a key pathophysiological feature of metabolic syndrome (25). Previous studies have associated metabolic syndrome with AP, and recent genetic evidence further supports a potential relationship between the two (26, 27). On a strict mechanistic level, such a relationship is driven by the fact that IR may accelerate lipolysis, elevate circulating FFAs, and promote hepatic TG synthesis, thereby exacerbating hypertriglyceridemia and lipid metabolic disorders (28, 29). These alterations may subsequently contribute to the onset and recurrence of HAP. In addition, IR and dysregulated lipid metabolism may mutually exacerbate each other, forming a vicious cycle. This interplay may partly explain the persistently high recurrence risk observed in patients with HAP from a pathophysiological perspective.

A previous study has reported that IR independently affects the prognosis of AP (30). Mechanistically, IR can promote MCP-1 expression and inhibit the insulin–mTORC2 pathway, thereby contributing to chronic inflammation in adipose tissue (31). In AP, inflammation of pancreatic and peripancreatic adipose tissue is considered a critical driver of disease pathogenesis (32). Currently, the primary mechanisms underlying HAP include the lipotoxic effects of elevated serum FFAs and dyslipidemia-induced increases in plasma viscosity, which can lead to microcirculatory disturbances (33). IR may further exacerbate these pathogenic processes. Specifically, elevated serum FFAs can intensify IR, while IR may in turn promote further increases in circulating FFAs (34). Therefore, IR-mediated metabolic disturbances may play a pivotal role in HAP recurrence. However, the precise mechanisms underlying this association warrant further investigation.

Our study provides a comprehensive evaluation of various IR-related indices in predicting HAP recurrence, yielding several key insights. First, we confirmed that elevated levels of TyG-BMI, TG/HDL-C, and METS-IR are independent risk factors for disease relapse. Crucially, TyG-BMI demonstrated exceptional robustness, maintaining its predictive value after FDR correction and exhibiting a clear, approximately linear dose-response relationship with HAP recurrence. Furthermore, the discordant NRI and IDI outcomes highlight that these biomarkers possess distinct, complementary prognostic advantages. While TyG-BMI is superior in clinical risk reclassification, alternative markers may offer better probability discrimination, suggesting that a multi-index approach could optimize risk stratification in clinical practice.

Elevated IR-related indices have also been linked to poor prognosis in previous studies. Specifically, a high TyG index has been associated with the highest risk of mortality among patients with sepsis (35). Similarly, elevated TyG index levels have been strongly linked to poor prognosis in HAP (36). Higher TG/HDL-C levels have also been closely associated with poor prognosis or increased disease severity in HAP (37, 38). Consistent with these findings, our results suggest that HAP patients with elevated IR-related indices, especially those with a higher TyG-BMI, may require closer follow-up and more individualized clinical management. Nevertheless, the mechanisms linking IR-related metabolic abnormalities to HAP recurrence remain unclear and warrant further investigation.

Strengths of this study include the comparative evaluation of multiple routine surrogate indices of insulin resistance using multidimensional metrics. However, several limitations should be acknowledged. First, the retrospective design and the use of telephone follow-up for some recurrences occurring outside the participating centers may have introduced residual confounding and information bias. Second, although restricting the cohort to mild HAP helped maintain clinical homogeneity and reduce confounding from metabolic derangements caused by severe stress, it limits the generalizability of our findings to patients with moderate or severe disease. Third, the analyses were based only on surrogate indices measured at the index HAP episode. These indices are dynamic and may be altered during follow-up by lipid control treatment, glucose control therapy, dietary modification, weight control, and treatment adherence. Because detailed longitudinal treatment information and repeated laboratory measurements were not systematically available, these factors could not be incorporated into the models, and paired comparisons between the index and recurrent HAP episodes could not be reliably performed. This may have resulted in confounding that changed over time and misclassification of exposure status. Therefore, our findings should be interpreted as baseline prognostic associations rather than causal effects or effects of metabolic exposure during follow-up. Finally, the modest discriminatory performance and the lack of external validation across broader populations warrant cautious interpretation and require confirmation in multicenter prospective studies.

## Conclusions

Surrogate indices related to IR were independently associated with increased recurrence risk in patients with mild HAP. While TyG-BMI demonstrated a consistent epidemiological association and specific advantages in risk reclassification, alternative markers provided complementary value in probability discrimination. These indices may serve as practical, dimension-specific supplementary biomarkers for baseline risk assessment.

## Data Availability

The original contributions presented in the study are included in the article/supplementary material, further inquiries can be directed to the corresponding authors.
